# Congenital duplication of the urethra with urethral diverticulum: a case report

**DOI:** 10.12688/f1000research.3848.1

**Published:** 2014-05-01

**Authors:** Darshan H Shah, Arvind P Ganpule, Ravindra B Sabnis, Mahesh R Desai

**Affiliations:** 1 Department of Urology, Muljibhai Patel Urological Hospital (MPUH), Nadiad, India

## Abstract

Duplication of the urethra is a rare congenital anomaly. Urethral duplication with the presence of diverticulum is a rare combination and to the best of our knowledge has  not been previously reported. We report a case of a 16 month old male child with duplication of the urethra and diverticulum arising from the ventral urethra. We also cover the intricacies and challenges in the management of such a case.

The opening of the narrowed accessory dorsal urethra at the verumontanum was cauterized and gradually the dorsal urethra became atrophied. The ventral urethral diverticulum was excised. This case is unique due to:

- The unusual presentation of swelling over the dorsum of the penis, together with duplication of the urethra with diverticulum.
- The use of cauterization as a treatment modality. Cauterization of the ventral urethra with a Bugbee electrode and diverticulectomy was performed. A glidewire helped in identifying the small opening of the dorsal urethra at the level of the verumontanum.

 The case also highlights the importance of endoscopic management of this clinical entity.

## Introduction

Duplication of the urethra is a rare congenital anomaly. Most cases involve incomplete duplication of the urethra. This anomaly is more common in males ^1^. The etiology of urethral duplication is unclear, no hypothesis explains the basis for all cases ^2, 3^. Urethral duplication with the presence of a diverticulum is a rare combination and to the best of our knowledge has not been previously reported.

In this report we describe a rare case of urethral duplication presenting as a urethral diverticulum in the ventral urethral passage, whilst the dorsal opening was abnormal. We also allude to the intricacies and challenges in the management of such a case.

## Case report

**Figure 1.**
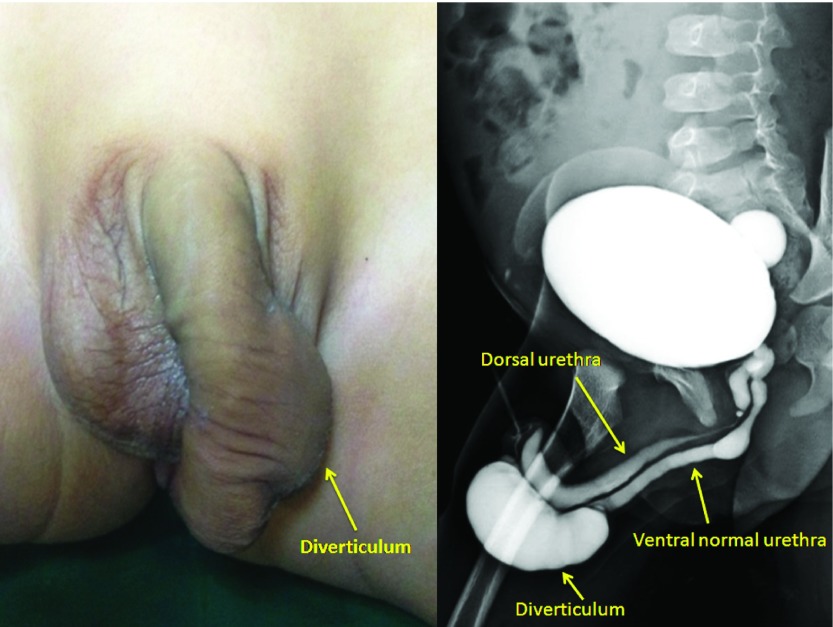
**1A**) Clinical photograph showing diverticulum at the distal part of the penis. **1B**) Micturating cystourethrogram showing complete duplication of the urethra in the sagittal plane. Large diverticulum arising from distal part of ventral urethra with small bladder diverticulum.

A 16 month old male child from India with duplication of the urethra and a diverticulum arising from ventral urethra was presented at our clinic in 2013. He presented with swelling of the penis, which increased in size with urination. General examination revealed an otherwise healthy child. Local examination revealed an approximately 4×2cm sized soft tissue swelling on the ventral aspect of the distal part of penis, cystic in nature ( Figure 1A). The meatus at the tip of the glans (dorsal urethral opening) of the penis was tiny and admitted a no. 22 G Intracath tip. On compression, drops of urine egressed from the meatal opening. Another meatal opening (ventral urethral opening) was seen 10mm proximal and ventral to first opening, which was wide and admitted a 10fr (3.33mm) infant feeding tube. A micturating cystourethrogram (MCUG) showed complete duplication of the urethra with a diverticulum arising from ventral urethra near its terminal part with a small para ureteral bladder diverticulum ( Figure 1B).

**Figure 2.**
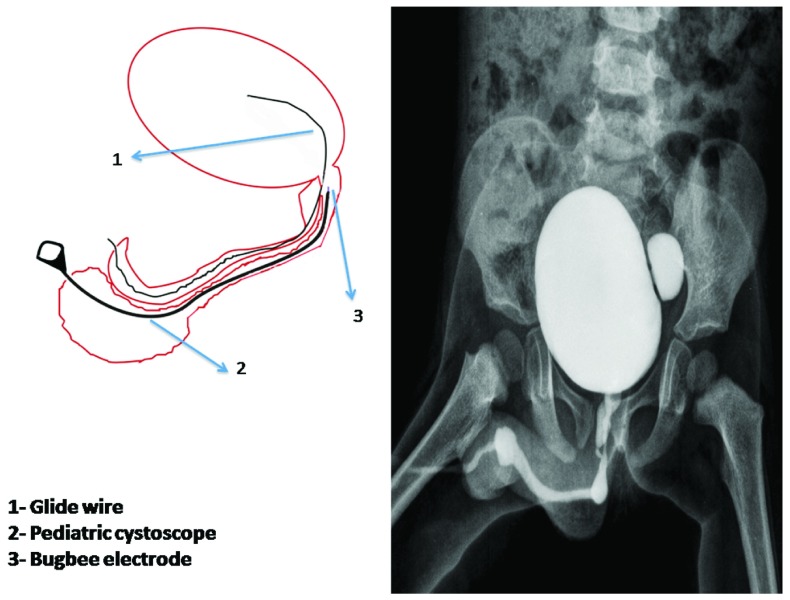
**2A**) Pictorial diagram showing the glidewire passed from the dorsal urethra to identify its opening into the normal ventral urethra. The cystoscope was passed from the ventral urethra up to the opening of dorsal urethra with a Bugbee electrode for fulguration of this opening. **2B**) Post-operative micturating cystourethrogram showing normal urethra, no urethral diverticulum and complete obliteration of the dorsal urethra is noted.

Cytsoscopy was done with a 9.5Fr cystoscope (KarlStorz, Germany). A Bugbee electrode, 3fr (KarlStorz, Germany) that could pass through a 3fr working channel of the cystoscope was used. Cystoscopy revealed a normal ventral urethra with a diverticulum. The dorsal urethra was abnormal and was narrowed in the proximal part. Its opening into the normal ventral urethra was localized with difficulty after passing a 0.025”/0.64mm glidewire (Terumo Corporation, Tokyo-Japan) through it ( Figure 2A). The opening was just proximal to the verumontanum at 10 o’clock. The dorsal urethra was cauterized at its opening into the ventral urethra using a Bugbee electrode and open diverticulectomy of the ventral urethral diverticulum was performed.

The patient was doing well at 6 months follow up with a good urinary stream ( Figure 2B).

## Discussion

Duplication of the urethra can occur with complete duplication of the penis or urinary bladder in the most extreme cases ^4^. Urethral duplication may be sagittal or collateral. In our case it was sagittal. Sagittal duplication takes the form of two channels running one above the other in the sagittal plane, whereas in the collateral form, the duplicate urethras run side by side. Most urethral duplications occur in the sagittal plane within a single penis and most are incomplete. Usually in such cases the ventral urethra is the dominant one ^5^. The most common sagittal variety is an orthotopic principal urethral channel and an epispadiac accessory urethra lying dorsal to it.

There are several different classifications describing urethral duplications. The classification by Effman *et al.* is the most widely used ^6^. According to this classification; the present case was type II A-2 (complete urethral duplication with the second urethra arising from first one and coursing independently into separate meatus).

Clinical presentation varies from type to type. Double urinary stream is one of the presentations of urethra duplication and may be bothersome when the ventral meatus is too proximal over the penis. Presentation may also include repeated urinary tract infections, incontinence or it may be asymptomatic and the only concern being a double meatus ^7^. This happens particularly when both meatus are very nearby.

Clinical examination and retrograde urethrogram (RGU) with MCUG should be sufficient for diagnosis in most cases. However, sonourethrograms and magnetic resonance imaging (MRI) are now also being used as adjunct procedures. Both will give excellent soft tissue details such as plaque or calcification which are associated with chordee in such cases ^8^.

Detailed knowledge of urethral duplication is important when planning for any surgical procedure for its correction. Many patients are asymptomatic and do not require any surgery. Indications for surgery are bothersome symptoms and cosmetic or functional deformity. Surgical reconstruction varies from case to case. It may range from simple meatoplasty to complex staged urethroplasty, depending on the severity of case. Most procedures involve excision of the accessory urethra with reconstruction of the dominant urethra ^9^. A favorable outcome is achieved in most of cases after reconstructive surgery. Dilatation of the orthotopic urethra is s more controversial option ^10^. Holst *et al.* have described fulguration of an atypical urethra as another treatment option ^11^.

Our treatment technique in this case was unique (minimally invasive) and successful. The opening of narrowed accessory urethra at the verumontanum was cauterized and the dorsal narrow accessory urethra gradually atrophied and had disappeared at a 6 month follow up MCUG. The ventral urethral diverticulum was excised at same time. This approach was chosen, given the concerns of infertility and incontinence associated with excision of such a long abnormal urethral tract ^5^.

In summary, the uniqueness of our case lies in the following facts:

– Unusual presentation of swelling over the dorsum of the penis, duplication of urethra with diverticulum.
– Cauterization was used as a treatment. Cauterization of the ventral urethra with a Bugbee electrode and diverticulectomy was offered as a treatment modality. The glidewire helped in identifying the small opening.

The case also highlights the importance of endoscopic management of this clinical entity.

## Consent

Before surgical procedure written informed consent obtained from patient’s parents. Written informed consent for publication of clinical details and clinical images was also obtained.
