# Supplementary material for: Congenital duplication of the urethra with urethral diverticulum: a case report
Source: F1000Res. 2014 May 1;3:99. [Version 1] doi: 10.12688/f1000research.3848.1 (PMC4168746; doi:10.12688/f1000research.3848.1)
Supplement: Peer review history

# Peer review: Referee response for version 1

**DOI:** 10.5256/f1000research.4121.r5660

**Published:** 2014-08-20

**Authors:** Tamsin Greenwell

**Affiliations:** 1 Institute of Urology, University College London Hospital, London, UK

This is a nicely written case report of a novel treatment of an unusual problems and deserves indexing.

I have read this submission. I believe that I have an appropriate level of expertise to confirm that it is of an acceptable scientific standard.

# Peer review: Referee response for version 1

**DOI:** 10.5256/f1000research.4121.r5659

**Published:** 2014-08-08

**Authors:** Sanjay Kulkarni, Pankaj Joshi

**Affiliations:** 1 Department of Urology, Centre for Reconstructive Urethral Surgery, Pune, Maharashtra, India; 2 Department of Urology, Centre for Reconstructive Urology, Pune, Maharashtra, India

This is a good case report.

It highlights the fact that urethral duplication has varied presentations.

Ventral divertculum actually helped in diagnosing the patient early as it was a symptomatic cosmetic presentation.

The authors are highly skilled in Endourology and skillfully performed the cauterization of the dorsal urethra.

This suggests that minimally invasive endourological technique can be used for treatment of duplication.

The follow-up MCU shows excellent result.

We have read this submission. We believe that we have an appropriate level of expertise to confirm that it is of an acceptable scientific standard.

# Peer review: Referee response for version 1

**DOI:** 10.5256/f1000research.4121.r5280

**Published:** 2014-08-01

**Authors:** Ali Tourchi

**Affiliations:** 1 Division of Pediatric Urology, The Johns Hopkins Medical Institutions, Baltimore, MD, USA

The case reported in this paper is an extremely rare presentation of urethral duplication which led me to read it three times. Urethral duplication is a rare congenital finding in pediatric urology. Most patients with this anomaly are asymptomatic. When they are symptomatic, they present with a double urinary stream (the most common presentation), urinary tract infection, incontinence, epididymitis, and bladder outflow obstruction. The authors have not described the type of duplication very well and the reader will be confused whether it is a complete, Y-type or some other type of duplication. Most pediatric urologists and surgeons agree upon identifying the functional urethra prior to any surgical correction of this anomaly. The functional urethra can be determined by urodynamic studies of both urethrae but it was not done in this study. I humbly appreciate the authors performance of  cauterization of the ventral urethra; however, the remnant of the ventral urethra is observable on post-operative MCUG. This remnant is concerning for its potential to cause urinary tract infection or incontinence in the future.

I have read this submission. I believe that I have an appropriate level of expertise to state that I do not consider it to be of an acceptable scientific standard, for reasons outlined above.
